# Getting cells into shape by calcium-dependent actin cross-linking proteins

**DOI:** 10.3389/fcell.2023.1171930

**Published:** 2023-03-21

**Authors:** Franziska Lehne, Sven Bogdan

**Affiliations:** Department of Molecular Cell Physiology, Institute of Physiology and Pathophysiology, Philipps-University Marburg, Marburg, Germany

**Keywords:** actin-binding protein (ABP), crosslinker, calcium, EF-hand, *α*-actinin, plastin/fimbrin, EFhd2/Swiprosin-1, wound healing

## Abstract

The actin cytoskeleton represents a highly dynamic filament system providing cell structure and mechanical forces to drive a variety of cellular processes. The dynamics of the actin cytoskeleton are controlled by a number of conserved proteins that maintain the pool of actin monomers, promote actin nucleation, restrict the length of actin filaments and cross-link filaments into networks or bundles. Previous work has been established that cytoplasmic calcium is an important signal to rapidly relay information to the actin cytoskeleton, but the underlying mechanisms remain poorly understood. Here, we summarize new recent perspectives on how calcium fluxes are transduced to the actin cytoskeleton in a physiological context. In this mini-review we will focus on three calcium-binding EF-hand-containing actin cross-linking proteins, *α*-actinin, plastin and EFHD2/Swiprosin-1, and how these conserved proteins affect the cell’s actin reorganization in the context of cell migration and wound closure in response to calcium.

## Introduction

Filamentous actin (F-actin) is a dynamic polymer providing mechanical forces to change cell morphology. The dynamic rearrangements and turnover of F-actin are pivotal corner stones for cellular functions such as intracellular transport, cytokinesis and cell migration. F-Actin polymerization and depolymerization are controlled by different families of actin binding proteins (ABPs). Profilin and sequestering thymosin-β4 compete for binding of actin monomers thereby modulating the concentration of free actin monomers available for polymerization (Pollard, 2016). Nucleation promoting factors (NPF) such as the WASp/SCAR family proteins at the cell membrane are activated through small Rho GTPases and can, in turn, activate the actin-related protein (Arp)-2/3-complex (Blanchoin et al., 2000). Growth of a filament is terminated by capping proteins and eventually filaments are disassembled through severing and debranching by ADF/cofilin. Severing exposes filament barbed ends that can continue growing and the dissociated actin monomers can enter a new actin polymerization cycle (Pollard and Borisy, 2003). Furthermore, actin filaments are incorporated into different μm-scale higher order structures: from loosely cross-linked isotropic networks to tight and highly organized bundles (Svitkina, 2020). While F-actin bundles are found in the cell cortex, in thin finger-like cell protrusions such as filopodia and are also prominent in contractile actomyosin-rings during cytokinesis and wound closure, cross-linked actin networks are important for the broad lamellipodium of a migrating cell.

More than 23 different classes of ABPs have been shown to crosslink or bundle F-actin (Schliwa, 1994; Tseng et al., 2005). Several studies suggest that actin crosslinking proteins have redundant roles (Rivero et al., 1999; Lieleg et al., 2010), which allows for finely adjusting the mechanical and viscoelastic F-actin network properties through cooperation and competition. Some proteins frequently also exhibit a dual activity by either cross-linking filaments into loose networks or laterally into tight bundles of filaments *in vitro*, often depending on protein concentration and solution crowding (Park et al., 2021). Interestingly, some ABPs are regulated by calcium ions (Ca^2+^) which allows to coordinate rapid actin remodeling driving morphological changes in response to diverse environmental stimuli (Chun and Santella, 2009; Izadi et al., 2018; Bootman and Bultynck, 2020). Prominent calcium-regulated ABPs are EF-hand containing proteins such as *α*-actinin (Burridge and Feramisco, 1981; Noegel et al., 1987) and fimbrins/plastins (Lin et al., 1988; de Arruda et al., 1990; Lin et al., 1993b) and EFHD/Swiprosin (Dutting et al., 2011; Mielenz and Gunn-Moore, 2016). Thus, the study of Ca^2+^ binding proteins in the context of actin cytoskeleton reorganization is important for our understanding of fundamental processes such as cell shape changes in cell migration and epithelial wound closure. Advances in biochemical structural analysis and *in vivo* live-cell microscopy have revealed new perspectives, how Ca^2+^ can regulate dynamic actin cytoskeletal changes by acting on ABPs. This review aims to outline recent findings on Ca^2+^ regulation of the most prominent actin cross-linking proteins *α*-actinin, plastin and EFHD2/Swiprosin-1.

### Calcium-an emerging intracellular messenger

For the rapid relay of a Ca^2+^ stimulus, Ca^2+^-regulated proteins can either bind to Ca^2+^/calmodulin (CaM) or bind Ca^2+^ directly through calmodulin-like domains (CaMD) to induce specific Ca^2+^-dependent processes (Nakayama and Kretsinger, 1994; Chin and Means, 2000). Calmodulin is a highly conserved Ca^2+^ sensor protein that contains the common Ca^2+^ binding motif composed of paired helix-loop-helix EF-hands. Binding of Ca^2+^ to the EF-hand domains leads to a conformational change of the target protein (Tufty and Kretsinger, 1975). EF-hand containing proteins with CaMDs comprise one of the largest protein family with a wide range of biological functions that bind Ca^2+^ with different affinities ranging from 10^–6^ M to 10^–3^ M (Bagur and Hajnoczky, 2017; Kawasaki and Kretsinger, 2017). Over twenty subfamilies have been defined by both evolutionary history and functions (Kawasaki and Kretsinger, 2017). Among them, three prominent EF-hand subfamily groups contain Ca^2+^-regulated ABPs including the Fimbrin/plastin group (FIMB), the *α*-actinin group (ACTN) and the EFHD2/Swiprosin group (EFHD2_DM) (Kawasaki and Kretsinger, 2017).

Moreover, Ca^2+^ can act indirectly on ABPs through activation of Ca^2+^-dependent kinases and small Rho GTPases. Therefore, maintaining cellular Ca^2+^ homeostasis is crucial for cytoskeletal dynamics. For the effectiveness of Ca^2+^ flux into the cytoplasm, an approximately 20.000-fold gradient between intracellular and extracellular Ca^2+^ concentration must be maintained (reviewed in Clapham, 2007). Special pumps like the plasma membrane Ca^2+^ ATPases (PMCA) or the sarcoendoplasmic reticular Ca^2+^ ATPases (SERCA) constantly remove Ca^2+^ under energy expenditure from the cytoplasm to the extracellular space or the endoplasmic reticulum (ER) which acts as an intracellular Ca^2+^ storage (Clapham, 2007). Ca^2+^ from the ER can also be transferred to mitochondria, dynamic organelles that have been shown to be important in locally releasing Ca^2+^ pulses to induce cytoskeletal rearrangements during migration and wound healing (reviewed in Paupe and Prudent, 2018). Moreover, influx of extracellular Ca^2+^ through Ca^2+^-permeable ion channels at the plasma membrane can be regulated by the actin cytoskeleton itself (Qian and Xiang, 2019).

In migrating cells there is a tightly controlled gradient of Ca^2+^ concentration from low at the leading edge to high at the cell rear. The low background level at the front of the cell permits even small changes in Ca^2+^ concentration to relay signals efficiently allowing, for example, small Ca^2+^ pulses to regulate cycles of local lamellipodia retraction and adhesion (Brundage et al., 1991; Wei et al., 2009; Tsai and Meyer, 2012; Wei et al., 2012; Tsai et al., 2015). Furthermore, tissue damage in a continuous epithelial sheet will cause an efflux of Ca^2+^ ions from the damaged cells into the extracellular environment which are then rapidly transported by mechanosensitive ion channels into the cells surrounding the wound site (Nakamura et al., 2018). This signal is then propagated within the epithelial tissue by gap junctions between neighboring cells (Razzell et al., 2013). The immediate increase of intracellular Ca^2+^ concentration leads to the massive cytoskeletal remodeling important for effective wound healing such as contractile ring and membrane protrusion formation at the wound margin (Antunes et al., 2013). But the underlying molecular mechanisms how Ca^2+^ signals result in local changes in cell shape and cell behavior are still poorly understood.

### Calcium-dependent actin cross-linking proteins–Bridging the gap

**FIGURE 1:**
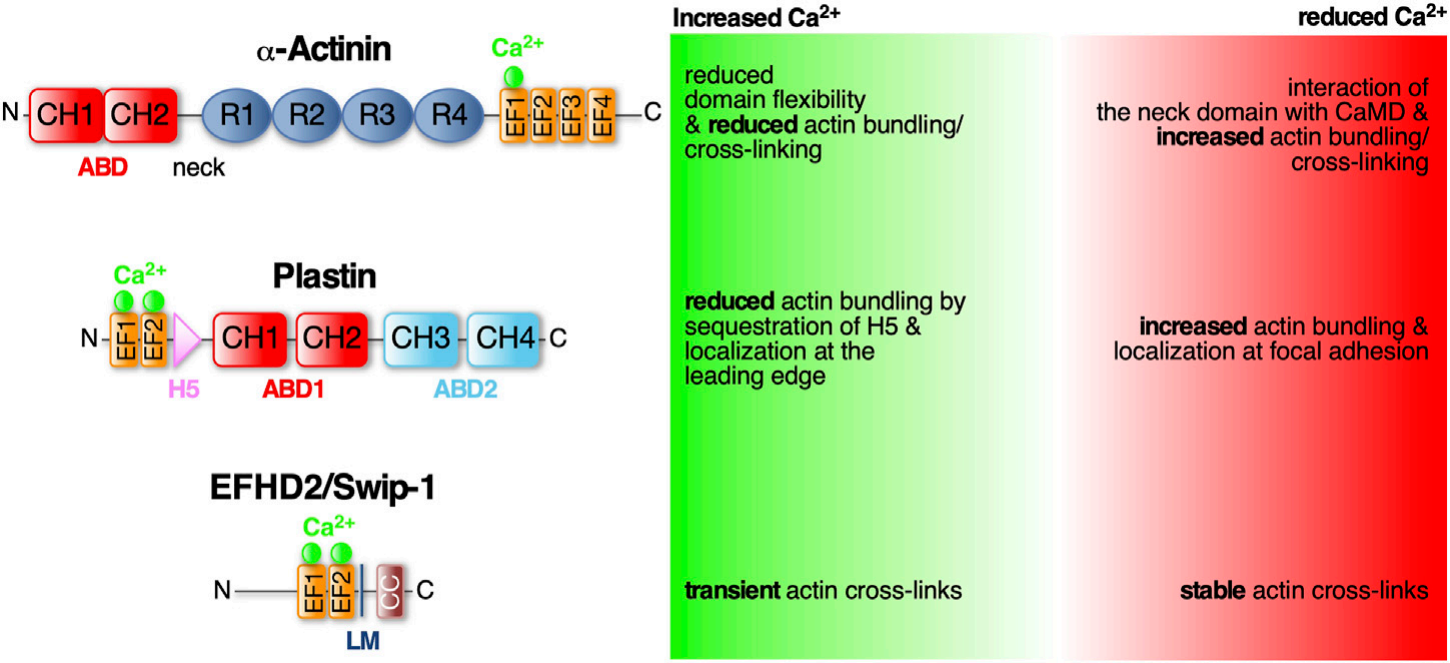
*α*-Actinin, Plastin and EFHD2/Swiprosin-1—three conserved EF- hand containing actin cross-linking proteins regulated by Ca^2+^. Schematic diagram of the protein domains of *α*-Actinin, Plastin and EFHD2/Swiprosin-1 and structural and functional changes in response to calcium. Domains as indicated: ABD (actin bindind domain) consists of two CH (calponin homology) domains; EF-hands (helix-loop-helix motif) that bind Ca^2+^ (green spheres); R (spectrin-like repeats) facilitate dimerization of *α*-Actinin; LM (ligand mimic domain); CC (coiled-coil domain) facilitates dimerization of EFHD2/Swiprosin-1; H5 (fith helix).

Calcium is a prominent regulatory cell signal which has also multiple effects on the structure and dynamics of the actin cytoskeleton (Chun and Santella, 2009; Izadi et al., 2018; Bootman and Bultynck, 2020). For the mechanical integrity of the actin cytoskeleton, overlapping actin filaments need to be attached to one another to rigidify and generate a stable F-actin network (Ydenberg et al., 2011; Svitkina, 2018; Svitkina, 2020). In the following we will discuss three highly evolutionarily conserved ABPs with Ca^2+^-regulated actin cross-linking activity (Figure 1).

### α-Actinin

α-Actinin belongs to the spectrin protein superfamily that dimerizes through four spectrin-like repeats (SLR) in their rod-shaped center (Figure 1). The central rod is connected on one side *via* a flexible neck region to the N-terminal actin binding domain (ABD), which is composed of two adjacent calponin homology domains (CH), and at the other side to a C-terminal CaMD, which is composed of two pairs of EF-hands (EF1/2 and EF3/4; see Murphy and Young, 2015). Antiparallel dimerization positions the terminal ABD and CaMD of opposing monomers at either end and in close proximity to each other allowing Ca^2+^-mediated regulation of the ABD (Djinović-Carugo et al., 1999; Sjöblom et al., 2008). The Ca^2+^ regulation has recently been shown to merely involve one single Ca^2+^ ion bound to the first EF-loop (Figure 1; Drmota Prebil et al., 2016; Backman, 2015). This domain is also affected by alternative splicing of the *α*-actinin gene which results in four different variants: Ca^2+^-insensitive muscle *α*-actinins and Ca^2+^-sensitive non-muscle *α*-actinins (Burridge and Feramisco, 1981; Sjöblom et al., 2008). In muscles, *α*-actinin is the predominant ABP cross-linking thin filaments from adjacent sarcomeres (Foley and Young, 2014). Here, Ca^2+^-independent cross-links are important for maintaining proper sarcomere structure during Ca^2+^-triggered muscle contraction. Instead, muscle *α*-actinins are regulated by phosphoinositides (Fukami et al., 1992). In non-muscle tissues, *α*-actinins are mainly involved in anchoring F-actin to cell-cell and cell-matrix adhesions (Foley and Young, 2014). In humans there are four *α*-actinin isoforms (1–4) with isoforms 1 and 4 widely expressed in non-muscle tissues (Murphy and Young, 2015). Mutations in the EF-hands of *α*-actinin-1 have been linked to congenital macrothrombocytopenia (CMTP), a rare blood disorder characterized by a reduced platelet count and increased platelet size due to aberrant actin cytoskeletal organization (Murphy and Young, 2015).

Human non-muscle *α*-actinin-1 mainly localizes to stress fibers and focal adhesion sites. Its actin cross-linking activity is inhibited by cytoplasmic Ca^2+^ concentrations above 0.1 mM (Burridge and Feramisco, 1981). Recently it has been shown that upon Ca^2+^ binding to the first of four EF-loops (EF1) in the CaMD, the local domain flexibility is altered leading to a more stabilized structure (Figure 1). It has been suggested that this modifies the CaMD interaction with the neck domain and therefore changing the ABD orientation in the dimer to effectively cross-link F-actin while actin binding is unchanged (Drmota Prebil et al., 2016). Similarly, Ca^2+^ has been described as allosteric regulator of F-actin bundling activity by modulating ABD orientational flexibility in *Entamoeba histolytica α*-actinin-2 (EhActn2) (Pinotsis et al., 2020). EhActn2 is similar to ancestral *α*-actinin containing only two SLR in the rod domain. Binding of Ca^2+^ was shown to support the interaction of the neck domain with EF-loops 3 and 4 thereby reducing the flexibility of a hinge region at the neck domain that allows ABD rotation around the rod axis. Interestingly, increasing the flexibility of the ABD also resulted in loss of actin bundling activity. Hence, Ca^2+^ binding to EhActn2 has been proposed to lead to an interdomain cross-talk increasing overall protein rigidity and therefore decreasing the conformational flexibility of the ABD which inhibits F-actin bundling (Pinotsis et al., 2020).

### Plastin/fimbrin

Plastins, also known as fimbrins, harbor two ABDs (Figure 1; ABD1 and 2) of tandem CH domains to effectively stabilize parallel F-actin bundles with a distance between adjacent filaments of approximately 120Å (Volkmann et al., 2001). There are three conserved plastin isoforms, namely I-, L-, and T-plastin (also called plastin 1-3, respectively) (Lin et al., 1993a), each encoded by a distinct gene and expressed in a tissue-specific manner. I-plastin is found in microvilli of intestinal and kidney epithelia (Lin et al., 1994) and in stereocilia of cochlear hair cells, where also T-plastin is expressed, though temporally restricted to stereocilia maturation (Daudet and Lebart, 2002). T-plastin is the most ubiquitously expressed isoform found in cells from solid tissues such as fibroblasts, endo- and epithelial cells but also neuronal cell such as microglia (Arpin et al., 1994; Shinomiya, 2012). L-plastin is expressed in hematopoetic cells serving fundamental functions in immunity including migration and adhesion (Morley, 2012). Further highlighting their importance for cell migration, L- and T-plastin are often associated with the invasiveness of metastatic cancer cells (reviewed in Lin et al., 1993b; Delanote et al., 2005).

The bundling activity of plastins is negatively regulated by Ca^2+^ binding. Plastins have two N-terminal EF-hands arranged in a so-called headpiece domain that is connected to ABD1 by a short 40 aa linker. Within this linker region lies a conserved fifth helix (H5) reminiscent of a calmodulin binding motif (Figure 1). This helix specifically interacts with the helices of the Ca^2+^-bound EF-hands. It has been suggested that in the tertiary protein structure of L-plastin, H5 acts as a wedge possibly between CH1 and CH2 of ABD1 changing their relative position and stabilizing an orientation that is favorable to actin binding (Ishida et al., 2017). The authors proposed that binding of Ca^2+^ to the EF-hands leads to sequestration of H5 from ABD1, destabilizing the orientation and thus decreasing actin bundling (Ishida et al., 2017). Another study looked at Ca^2+^ binding and its effect on actin binding in all three human plastin isoforms. They, however, identified ABD1 of L-plastin to bind F-actin Ca^2+^-independently while ABD2 is regulated by Ca^2+^ binding to the EF-hands (Schwebach et al., 2017). Therefore, the former hypothesis could still be applicable to CH3 and CH4 of ABD2. Another recent finding concerns the localization of T-plastin in osteoblast, osteocyte and fibroblast cells depending on Ca^2+^ binding. Wildtypic T-plastin can cycle between the lamellipodium and focal adhesions while Ca^2+^-hyposensitive T-plastin mutants localized exclusively to focal adhesions and Ca^2+^-hypersensitive mutants only in the lamellipodium. Chelation of Ca^2+^ led the latter to localize to focal adhesions as well, identifying Ca^2+^ binding as the regulator for T-plastin cycling between the leading edge and adhesion complexes. The authors proposed that in the presence of Ca^2+^, CH3 and CH4 of ABD2 are locked in an inhibited state by the headpiece domain therefore only ABD1 binds to F-actin. Binding of Ca^2+^ leads to detachment of the H5 from the EF-hands therefore releasing ABD2 from its structural constraints (Schwebach et al., 2020). However, the crystal structures of Ca^2+^-bound and -unbound human T-plastin has not been solved yet to reveal the exact inhibitory mechanism proposed for H5.

### EFHD2/Swiprosin-1

EFHD2/Swiprosin-1 (Swip-1) is also an EF-hand containing ABP that has recently been identified to create orthogonal F-actin cross-links aiding the rapid cytoskeletal rearrangements necessary for lamellipodia formation (Lehne et al., 2022). Physiologically, Swip-1 has been associated with a plethora of pathologies such as neurodegenerative diseases, acute and chronic inflammation and cancer especially invasive stages of malignant melanoma (Dütting et al., 2011; Mielenz and Gunn-Moore, 2016; Thylur et al., 2018). It has also been reported that Swip-1 is involved in BCR-induced Ca^2+^ flux controlling BCR signaling to activate B cells (Kroczek et al., 2010; Hagen et al., 2012) and in *Drosophila* embryos it was hypothesized that it regulates Ca^2+^-dependent exocytosis of electron dense vesicles during myoblast fusion (Hornbruch-Freitag et al., 2011).

**FIGURE 2:**
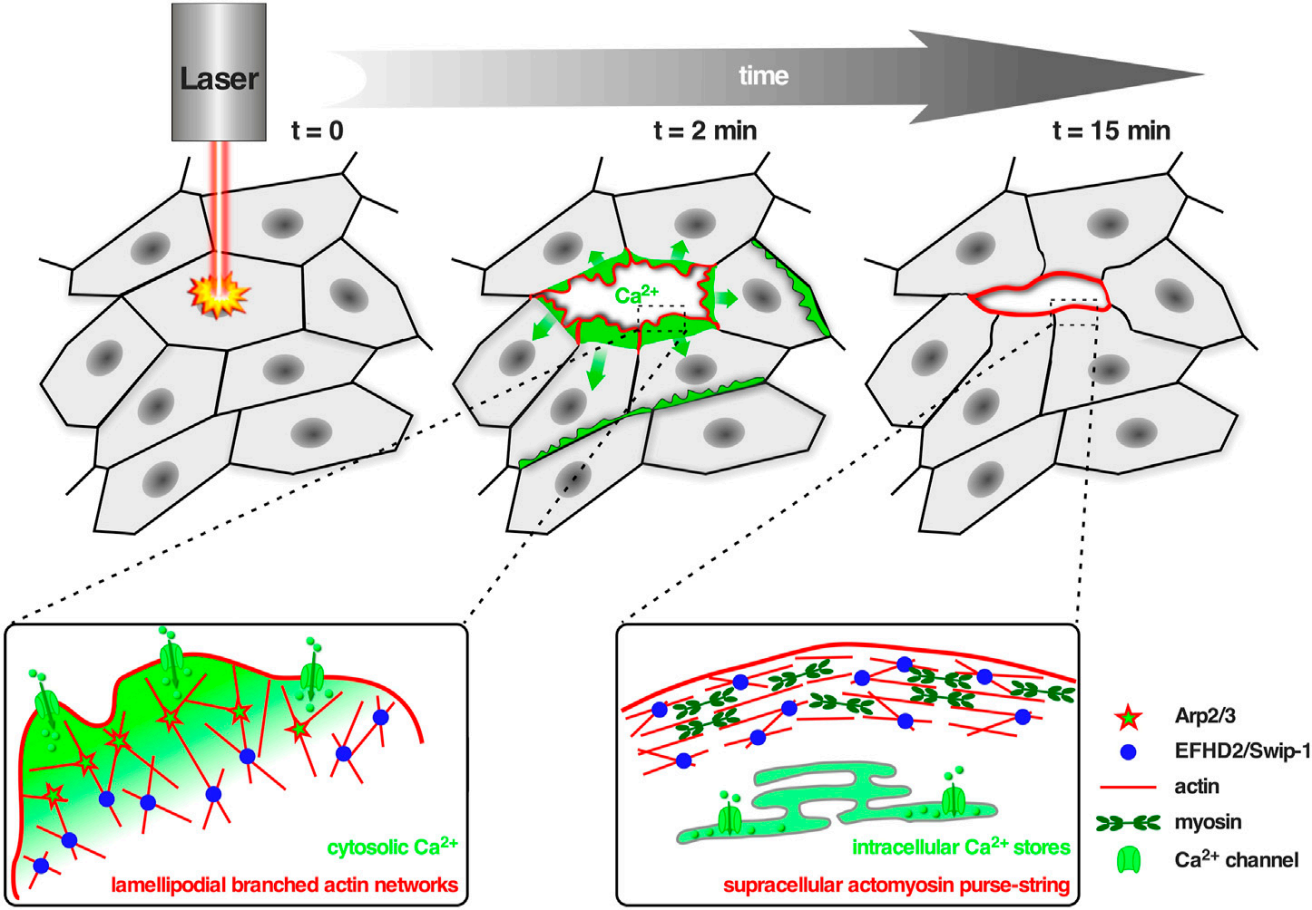
Swip-1 cross-linked lamellipodial actin networks drive single cell migration and epithelial wound closure. Upper panel: Schematic of the *in vivo* model to study calcium-dependent wound healing in the abdominal epidermis in *Drosophila*. Laser-induced single-cell ablation starts at *t* = 0 min. In the first two minutes (*t* = 2 min) F-actin assembled into broad lamellipodial protrusions within cells at the wound edge; lamellipodial protrusions reached a maximum size between 5–10 min after wounding. Later on (*t* = 15 min) lamellipodia formation decreased and a supracellular acto-myosin ring is formed at the leading edge of the wound, contracted laterally to pull cells forward and increasingly contributes to wound closure. Lower panel: Schematic showing the proposed role of Swip-1 in regulating lamellipodial actin networks and supracellular acto-myosin rings upon laser-induced single-cell wounding within the epidermis (see also text; adapted from Lehne et al., 2022). Left: The actin crosslinking activity of Swip-1 (blue spheres) synergizes with WRC-Arp2/3-branched actin nucleation (red stars) promoting generation of a stable and densely branched actin filament network. Plasma membrane damage or mechanical activation of calcium channels (green) allows a rapid reorganization of the actin cytoskeleton through Swip-1. Right: After initial calcium wave propagation, the levels of intracellular calcium decreased mediated by special pumps (green) like the sarcoendoplasmic reticular calcium ATPases (SERCA) which constantly remove calcium from the cytoplasm to the ER lumen. A supracellular actin-myosin ring is formed stabilized by stable actin cross-links induced by Swip-1.

Swip-1 contains a disordered region at the N-terminus that varies across species, two functional EF-hand domains, a connecting short *α*-helix called ligand mimic (LM) helix and a coiled-coil (CC) domain at its C-terminus allowing self-dimerization (Avramidou et al., 2007; Vega et al., 2008; Hagen et al., 2012; Ferrer-Acosta et al., 2013). After gene duplication in *Euteleostomi*, the *efhd2* gene was duplicated giving rise to the *efhd1* gene encoding EFHD1/Swiprosin-2 (Swip-2) (Dutting et al., 2011). While Swip-2 also binds Ca^2+^, it localizes in the inner mitochondria membrane and will therefore not be further discussed here. Although it is known that the cytoplasmically expressed Swip-1 binds F-actin (Huh et al., 2013; Kwon et al., 2013; Huh et al., 2015; Tu et al., 2018), the localization and number of ABDs has not been fully addressed, yet. Most probable, a binding site is located within the first EF-hand domain because its deletion has been shown to sufficiently dimmish F-actin binding (Moreno-Layseca et al., 2021). Swip-1’s role in cell motility and lamellipodia formation has been established (Huh et al., 2013; Kwon et al., 2013), but the mechanism how it regulates the underlying necessary F-actin rearrangements in response to Ca^2+^ has only recently been elucidated (Lehne et al., 2022). *In vivo* studies in *Drosophila* showed that Swip-1 is enriched in the forming lamellipodium of both migrating immune cells and at epidermal wound edges where it drives the initial phase of wound healing to re-establish tissue integrity (Lehne et al., 2022). In the absence of Ca^2+^, Swip-1-mediated cross-links stabilizes the F-actin network while Ca^2+^ binding to Swip-1 results in transient cross-links. Therefore, upon Ca^2+^ binding to Swip-1, the actin meshwork becomes relaxed making the filaments accessible for severing and branching (Figure 2). A model has been proposed in which increased Ca^2+^ concentrations reduce Swip-1 cross-linking activity to promote rapid reorganization of existing actin networks, to drive fast and efficient epithelial wound closure by extension of newly formed lamellipodia (Figure 2). Defective constriction of the wound margin in mutant epidermal cells further suggests that Swip-1 function might also be involved in the subsequent formation of the contractile supracellular acto-myosin ring forming along the wound margin (Figure 2; Lehne et al., 2022). Thus, Swip-1 could act as a Ca^2+^ sensor protein responding to the transient local changes of Ca^2+^ concentration at the leading edge of migrating cells and in wounded epidermis (Lehne et al., 2022).

Human Swip-1 also bundles efficiently actin filaments, however this activity is calcium-insensitive (Lehne et al., 2022). Of note here, Ca^2+^ concentrations of up to 1 mM did not completely abolish Swip-1-mediated cross-links but only shifted them from stable to transient. Structurally, binding of Ca^2+^ at the two EF-hands does not alter the overall secondary structure nor the actin-binding properties (Ferrer-Acosta et al., 2013). However, it is believed that upon depletion of Ca^2+^ the local conformation at the EF-hand domains and LM helix becomes more flexible (Park et al., 2016). Because recent findings have been contradictory in regards to positive or negative regulation of Swip-1 by Ca^2+^, the underlying structural changes and how it influences Swip-1 cross-linking activity are of great interest. Remarkably, a recent study also identified a calcium-independent role of Swip-1 in regulated exocytosis where it contributes to the recruitment of Rho-GTPase regulating actomyosin activity to drive proper vesicle membrane crumpling and expulsion of cargo (Lehne and Bogdan, 2023).

## Conclusion and future perspectives

Ca^2+^ is an important second messenger, whose change in concentration induces and controls spatiotemporally pathways of many physiological processes such as proliferation or cell death and many processes dependent on cell migration such as development, immune response or cancer metastases (Clapham, 2007; Evans and Falke, 2007; Tsai et al., 2015). The main pillars of cellular migration are protrusions, forming new adhesion sites allowing traction force generation and lastly release of the cell rear by dissolving mature adhesions (Lauffenburger and Horwitz, 1996). These processes rely on dynamic cellular changes where Ca^2+^-dependent actin cross-linkers can respond to the fast-acting second messenger. Low cytoplasmic Ca^2+^ concentration at the protruding edge would stabilize F-actin cross-links that can be rearranged by local Ca^2+^ pulses while high Ca^2+^ at the trailing edge destabilizes adhesion complexes (Drmota Prebil et al., 2016). It is therefore not surprising that aberrant Ca^2+^ signaling as well as the described cross-linkers, *α*-actinin, plastins and Swip-1, all have been implicated in migration-linked pathologies such as invasive cancers (Shinomiya, 2012; Huh et al., 2015; Tsai et al., 2015). A common theme seems to be that on a structural level, binding of Ca^2+^ influences protein domain flexibility through interdomain cross-talk. Further investigations like comparing Ca^2+^-bound and -unbound states can help elucidate the regulatory mechanisms of structural changes and possibly uncover new strategies for cancer treatment.

Not to be dismissed, actin cross-linkers inherently bind actin so other regulating mechanisms independently of their bundling activity are conceivable such as regulating actin turnover. For instance, Swip-1 has been implicated to regulate small Rho GTPases (Huh et al., 2015; Lehne and Bogdan, 2023) and has been shown to regulate actin depolymerization by recruiting cofilin (Huh et al., 2013). On the other hand, T-plastin has been suggested to control actin turnover by displacing cofilin therefore decreasing the actin depolymerization rate and possibly recruiting other ABPs such as *α*-actinin (Giganti et al., 2005). This also shows that functional cross-linker synergy poses interesting new ways of deciphering dynamic actin cytoskeleton regulation. In line with this, for future investigations and interpretation of obtained results one should also consider that the concentration of a cross-linking protein is crucial for its function (Lieleg et al., 2010). For example, *α*-actinin has been shown to cross-link actin at low but bundle it at high concentrations (Tseng et al., 2002; Lieleg et al., 2010). Thus, *in vitro* experiments are crucial to dissect how nanoscale structural changes of a cross-linking protein can alter the mesoscale geometric and mechanical properties of the actin cytoskeleton network. On the other hand, the limitations of *in vitro* studies should be emphasized. ABPs often interact with multiple proteins that integrate signaling pathways between the plasma membrane and the actin cytoskeleton in a living cell. Thus, structure-function *in vivo* studies using genetic model systems such as *Drosophila* will further improve our understanding of how the cross-linkers function in a physiological context.

## Abbreviations

ABD, actin binding domain; ABP, actin binding protein; Ca2+, calcium; CaMD, calmodulin-like domain; CH, calponin homology domains; F-actin, filamentous actin; SLR, spectrin-like repeats; Swip-1; EFHD2/Swiprosin-1.
